# Case Report: Placental site trophoblastic tumor revealed by a clinical pelvic abscess

**DOI:** 10.12688/f1000research.75075.1

**Published:** 2022-02-01

**Authors:** Souayeh Nesrine, Hajer Bettaieb, Wael Mbarki, Ben Brahim Ehsen, Helal Imen, Ben Nasr Mehdi, Oueslati Hedhili, Hsayaoui Najeh, Chaouki Mbarki

**Affiliations:** 1 Department of Obstetrics and Gynecology, Ben Arous Hospital, Faculty of Medicine, Ben Arous Hospital, El Manar, Tunisia; 2 Department of Pathology, Habib Thameur Hospital, Faculty of Medicine of Tunis, Hbib Thameur Hospital, El Manar, Tunisia

**Keywords:** Gestational Trophoblastic Disease, Placenta Diseases, Trophoblastic Tumor, Placental Site, pathology.

## Abstract

We report an uncommon clinical presentation of a placental site trophoblastic tumor. The patient presented initially with abdominal pain with, fever, bleeding and pelvic mass on ultrasonography leading to the wrong diagnosis of a pelvic abscess. Dilation and curettage were performed and pathological examination confirmed the diagnosis. of placental site trophoblastic tumor. Therefore, she underwent abdominal hysterectomy. Four years after surgery, the patient is still disease free. Gestational trophoblastic diseases should be considered in every patient presenting abnormal uterine bleeding after delivery or pregnancy loss despite the associated symptoms being very unusual.

## Introduction

Placental site trophoblastic tumor (PSTT) is a rare tumor, representing 1% of gestational trophoblastic diseases. ^1^^,^^2^ It mainly affects women of childbearing age after delivery or pregnancy loss. ^1^^–^^3^ In this paper, we report a case of placental site trophoblastic tumor diagnosed by an array of pelvic abscess associated with bleeding occurring two months after delivery. The evolution, after surgical treatment by hysterectomy, was favorable.

## Case report

We report the case of an unemployed 32-year-old Arabic woman, gravida 1 and para 1, without medical history, gave birth naturally and without complications to a full-term healthy newborn. Two months after her delivery, she consulted our department with persisting metrorrhagia associated with fever of 39°C. The patient’s examination revealed that the bleeding has been noted since the delivery day and it had increased two days earlier.

**Figure 1.**
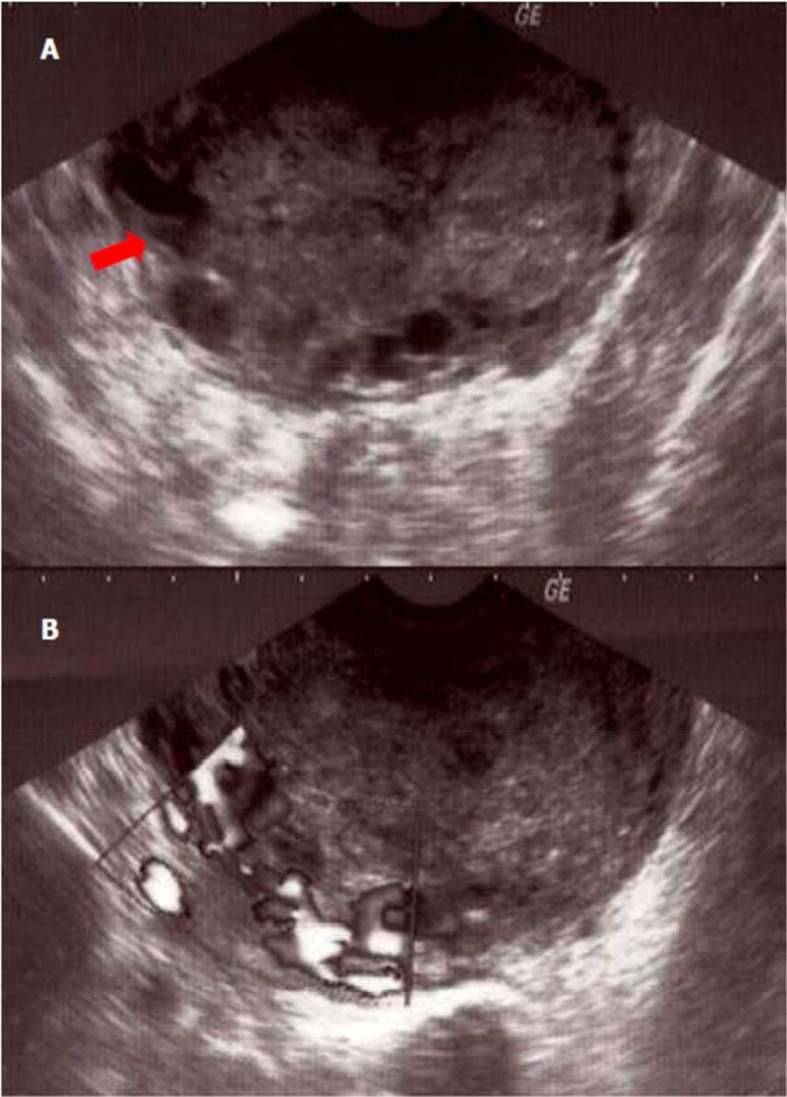
Sonographic appearance of the tumor of the placental site tumor. (A) Echogenic tumor occupying the uterine cavity (arrow) with infiltration of the myometrium; (B) appearance vascularized tumor at color Doppler.

A physical examination revealed a fever of 39.5°C and abdominal tenderness. The blood pressure was 110/70 mm Hg and the heart rate was 82 beats/mn. Furthermore, there was no specific digestive or urinary symptom. Per speculum examination, the cervix was enlarged with purulent vaginal discharge. On digital pelvic examination, the uterus was increased in size accessed at 12 week gestation and its mobilization was painful. Blood tests showed an inflammatory syndrome with a C-reactive protein at 200 mg/l [≤ 6mg/l] and white blood cells at 17000/mm ^3^ [4000-10000/mm ^3^]. The beta-human chorionic gonadotropin (β-HCG) rate was positive at 674 U/l [< 5U/l]. The transvaginal sonography showed a large sized uterus containing an echogenic heterogeneous endometrial mass, vascularized on Doppler examination. This process seemed to invade the myometrium in part. In addition, ultrasound showed an average effusion in the Douglas' pouch ( Figure 1) with a probable left adnexal abscess.

Laparoscopic adnexal drainage and endometrial curettage were performed. The infectious episode resolved promptly with antibiotics; gentamicin 160 mg per day for 5 days metronidazole 500 mg three times a day for 15 days and cefotaxim 1g three times a day for 15 days. Pathological examination of the endometrial curettage specimen reported a proliferation of large tumor cell with eosinophilic cytoplasm and nuclear atypia, infiltrating the myometrium. These cells were positive for pro inflammatory cytokine (IL6) in the immunohistochemistry studies and the final diagnosis was a placental site trophoblastic tumor.

**Figure 2.**
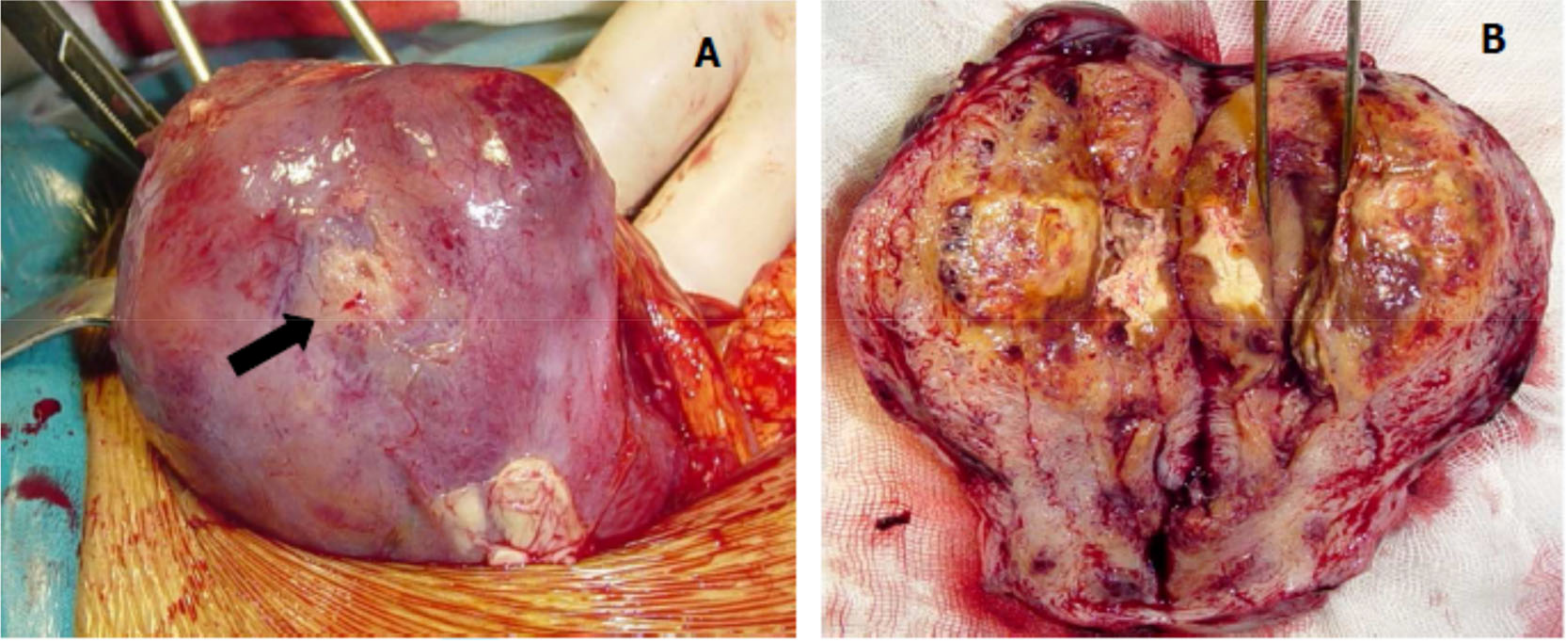
Macroscopic appearance of the placental site tumor. (A) Peri operative view showing increased uterine size, yellowish area seat (arrow); (B) appearance of the tumor to cut, yellowish tumor occupying the uterine cavity and infiltrating the myometrium.

**Figure 3.**
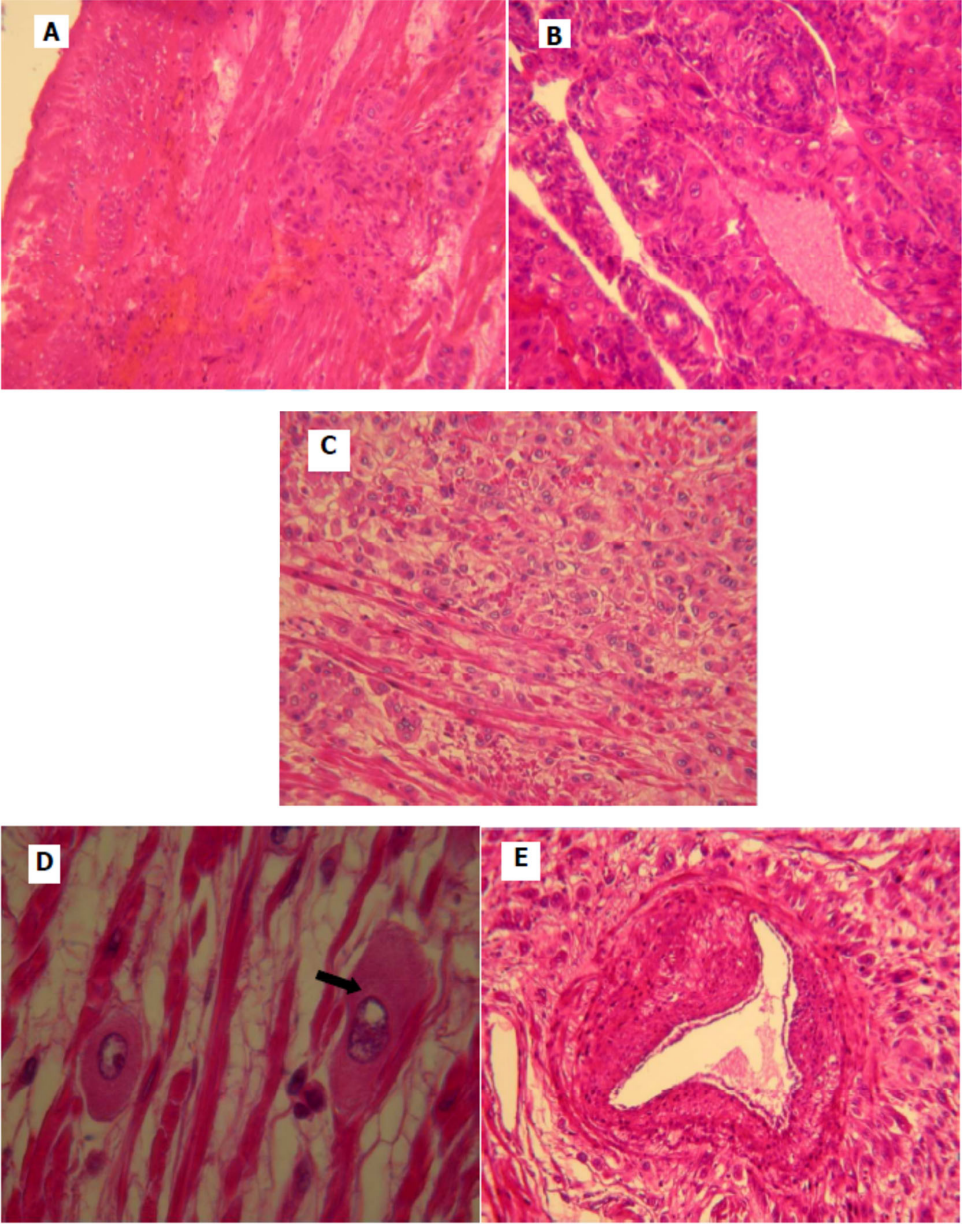
Histological appearance of the placental site tumor. (A) Monomorphic cell proliferation of intermediate trophoblast; (B, C) tumor cells infiltrate the myometrium form of clusters, dissociating smooth muscle bundles (B: low magnification, C: magnification); (D) proliferation of large cells with eosinophilic cytoplasm and lobulated nucleus; (E) home of tumor cells with the presence of vascular emboli.

A laparotomic hysterectomy with preservation of ovaries was performed. On gross examination there was a mass of 6 cm localized to the myometrium of bluish appearance with areas of hemorrhage and necrosis. The tumors had ill-defined borders and invaded into the myometrium ( Figure 2). Pathological examination was similar to that described in the curettage specimen, showing a proliferation of monomorphic intermediate trophoblasts ( Figure 3A). The tumor cells demonstrated nuclear atypia, eosinophilic cytoplasm ( Figure 3D) and myometrial invasion ( Figure 3B, C). Extensive necrosis, hemorrhage and vascular invasion are observed ( Figure 3E). The final pathology report confirmed the diagnosis of PSTT infiltrating the myometrium. The postoperative course was favorable. The patient had a regular follow-up every month for the first year combining a clinical examination and a serum level of β-HCG. She had a clinical check-up and pelvic ultrasound every year for two years, no sign of recurrence was noted. Four years after surgery, the patient is still disease free.

## Discussion

In 1895, Marchand first described PSTT and named it atypical chorioepithelioma. ^4^ In 1981, Scully and Young described the morphological details and recognized it as a neoplastic process, and named it PSTT. ^5^ PSTTs are a rare subtype of gestational trophoblastic disease, accounting for less than 1% of all cases. ^1^^,^^2^^,^^6^ It mainly affects women of childbearing age and it is seen in patients between 19-62 years, with an average age of 30 years. ^1^^–^^3^^,^^6^^,^^7^ PSTT can occur after a normal pregnancy, abortion, term delivery, ectopic pregnancy or molar pregnancy. ^1^^–^^3^^,^^6^^–^^8^ Here we reported a case of PSTT occurring after a normal pregnancy. This pathology may occur months to years after pregnancy with the average interval between the antecedent pregnancy and diagnosis being 16 to 18 months. ^1^^–^^3^^,^^6^^,^^9^ Diagnosis of PSTT can be difficult due to the nonspecific clinical signs. Abnormal uterine bleeding is the most common presenting feature. ^1^^–^^3^^,^^6^^–^^9^ Metrorrhagia can also be associated with amenorrhea. ^1^^,^^8^ The peculiarity of our case lies in the fact that the tumor was manifested by an array of pelvic abscess associated with bleeding; such clinical presentation was rare in literature. ^8^ On digital pelvic examination, the uterus can be enlarged or normal sized. ^2^^,^^8^ A wide range of other rare symptoms have also been reported, including galactorrhea, virilization, nephrotic syndrome, and polycythemia. ^2^^,^^8^ PSTT can also be revealed by metastasis. ^2^^,^^7^ The serum levels of β-hCG are usually in the range of 1000-2000 mIU/mL. ^1^^–^^3^^,^^6^^,^^9^ The origin of PSTT is the intermediate trophoblast cells which secrete little beta HCG and a lot of placental lactogenic hormone. ^6^^,^^9^

On gross examination, the tumors are located primarily in the endomyometrium, presenting as polypoid or nodular masses, with a variable diameter up to 10 cm. ^1^^,^^6^ The sectioned surfaces of the tumors are solid, often fleshy, and usually yellow with necrosis, and hemorrhage. ^1^^,^^2^^,^^6^ The tumor may extend into the cervix or infiltrate the serous, the adnexa or the round ligaments. ^2^ In our case, the tumor invaded the myometrium without reaching the serous. The diagnosis of PSTT is based on pathological examination.

On microscopic examination, we typically find a proliferation of intermediate trophoblastic cells without chorionic villi, which infiltrate muscle fibers. ^2^ Vascular invasion, necrosis and hemorrhage are also often observed. ^2^

The tumor is composed of a relatively monotonous population of polygonal cells with nuclear atypia and moderately abundant cytoplasm that can be amphophilic, eosinophilic or clear. ^1^^,^^2^ The distinctive pattern of vascular invasion and deposition of fibrinoid material are the key diagnostic features. ^1^^–^^3^ Usually, PSTTs are benign tumors. However, they have an unpredictable malignant potential and between 10 and 20% of patients have metastatic disease at the time of presentation. ^1^^,^^2^^,^^7^^,^^10^ Metastasis site include peritoneum, vagina, lung, liver and brain.

Hysterectomy without bilateral oophorectomy is the gold standard in PSTT management. ^2^^,^^10^ In fact, this entity is particularly less responding to chemotherapy than hydatiforme mole, invasive mole and choriocarcinoma. ^2^^,^^10^ When there is extension beyond the uterus, surgical treatment should be associated to chemotherapy. ^2^^,^^10^

Actually, the FIGO anatomical staging system is the strongest predictor of patient outcome presenting with PSTT. ^2^^,^^7^^,^^10^^,^^11^ After surgical treatment, patients should be followed up during years to optimize the chance of detecting a relapse or metastasis. ^7^^,^^10^^,^^11^

## Strengths and limitations

This case report is interesting since it’s the first reporting PSTT simulating a pelvic abscess. However, PTTT are rare which prevents researchers from realizing large scale studies in order to develop valid management protocols.

## Conclusion

Although PSTTs are extremely rare worldwide, the diagnosis should be considered during the post-partum and post-abortion periods if there is persistent metrorrhagia. After surgical treatment, the follow up is needed to be accurate, to help recognize a relapse or metastasis.

## Data availability

All data underlying the results are available as part of the article and no additional source data are required.

## Consent

Written informed consent for publication of clinical details and clinical images was obtained from the patient.
