# Supplementary material for: Case Report: Placental site trophoblastic tumor revealed by a clinical pelvic abscess
Source: F1000Res. 2024 Oct 21;11:129. Originally published 2022 Feb 1. [Version 3] doi: 10.12688/f1000research.75075.3 (PMC11499744; doi:10.12688/f1000research.75075.3)
Supplement: Peer review history

# Reviewer report: Reviewer response for version 3

**DOI:** 10.5256/f1000research.173598.r333450

**Published:** 2024-10-23

**Authors:** Guglielmo Stabile

**Affiliations:** 1 Department of Medical and Surgical Sciences, Institute of Obstetrics and Gynecology,, University of Foggia Departments of Medicine (Ringgold ID: 507873), Foggia, Foggia, Italy

The manuscript has been improved. The manuscript is suitable for indexing.

Are enough details provided of any physical examination and diagnostic tests, treatment given and outcomes?

Yes

Is the case presented with sufficient detail to be useful for other practitioners?

Yes

Is sufficient discussion included of the importance of the findings and their relevance to future understanding of disease processes, diagnosis or treatment?

No

Is the background of the case’s history and progression described in sufficient detail?

Yes

Reviewer Expertise:

Expert in Placental Pathology

I confirm that I have read this submission and believe that I have an appropriate level of expertise to confirm that it is of an acceptable scientific standard.

# Reviewer report: Reviewer response for version 3

**DOI:** 10.5256/f1000research.173598.r333452

**Published:** 2024-10-23

**Authors:** Seetu Palo

**Affiliations:** 1 All India Institute of Medical Sciences, Hyderabad, India

Although the authors have added a note on differential diagnoses, further refinement and correction is needed in this section. For example, the authors have mentioned that exaggerated placental site response (EPSR) is characterized by lack of nuclear atypia and invasive behavior, which is in contrast to existing literature. EPSR can exhibit certain degree of nuclear atypia and it is know to invade the superficial myometrium as well (Ref 1, Ref 2).

Are enough details provided of any physical examination and diagnostic tests, treatment given and outcomes?

Partly

Is the case presented with sufficient detail to be useful for other practitioners?

Yes

Is sufficient discussion included of the importance of the findings and their relevance to future understanding of disease processes, diagnosis or treatment?

Yes

Is the background of the case’s history and progression described in sufficient detail?

Yes

Reviewer Expertise:

Pathology

I confirm that I have read this submission and believe that I have an appropriate level of expertise to confirm that it is of an acceptable scientific standard, however I have significant reservations, as outlined above.

## References

[1] : Exaggerated placental site reaction detected during caesarean delivery: a case report. *Clin Exp Obstet Gynecol* .2012;39(2) :234-5 22905472

[2] : The pathology of intermediate trophoblastic tumors and tumor-like lesions. *Int J Gynecol Pathol* .2001;20(1) : 10.1097/00004347-200101000-00004 31-47 10.1097/00004347-200101000-00004 11192071

# Reviewer report: Reviewer response for version 2

**DOI:** 10.5256/f1000research.157888.r321955

**Published:** 2024-09-25

**Authors:** Seetu Palo

**Affiliations:** 1 All India Institute of Medical Sciences, Hyderabad, India

This is a well-documented case report on a rare condition, providing valuable insights into the diagnosis and management of placental site trophoblastic tumor (PSTT). Strengthening the discussion around the unique presentation and refinement in structure and depth of discussion, particularly around clinical implications and differential diagnosis, would improve the manuscript.

 The description of imaging findings is well-executed, and the figures provide useful visual aids. However, there are some issues with figure referencing and clarity. Figure 1 is briefly mentioned in the text, but the explanation could be expanded to better connect the ultrasound findings to the case narrative. Similarly, the histopathological micro-photographs, which forms the basis of diagnosis, need to be of better resolution with appropriate annotations for better understanding of the readers. The authors should discuss about microscopic differential diagnoses, such as, exaggerated placental site response, epithelioid trophoblastic tumor and gestational choriocarcinoma, and state how were these differentials excluded in this particular case.

 The description of the surgical management (hysterectomy) is clear, and the follow-up section is well done, providing a clear timeline of surveillance for recurrence, which is crucial in managing PSTT. However, additional information regarding β-HCG levels over time would further strengthen the post-treatment monitoring discussion. A brief note on emerging treatment options or potential prognostic factors could further enrich the discussion.

Are enough details provided of any physical examination and diagnostic tests, treatment given and outcomes?

Partly

Is the case presented with sufficient detail to be useful for other practitioners?

Yes

Is sufficient discussion included of the importance of the findings and their relevance to future understanding of disease processes, diagnosis or treatment?

Yes

Is the background of the case’s history and progression described in sufficient detail?

Yes

Reviewer Expertise:

Pathology

I confirm that I have read this submission and believe that I have an appropriate level of expertise to confirm that it is of an acceptable scientific standard, however I have significant reservations, as outlined above.

# Response

**Published:** 2024-10-16

**Authors:** chaouki mbarki

**Affiliations:** Ben arous hospital, Faculty of medecine of Tunis university Tunis EL MANAR, Tunisia

**Dear Reviewer,**

 Thank you for reviewing our article. I truly appreciate all your remarks and comments. I have made the necessary revisions, which you can find in the revised manuscript (version 3). I hope the updated version meets your approval.

 Thank you once again.

# Reviewer report: Reviewer response for version 2

**DOI:** 10.5256/f1000research.157888.r321960

**Published:** 2024-09-24

**Authors:** Guglielmo Stabile

**Affiliations:** 1 Department of Medical and Surgical Sciences, Institute of Obstetrics and Gynecology,, University of Foggia Departments of Medicine (Ringgold ID: 507873), Foggia, Foggia, Italy

Dear Authors and Editor, thank you to allow me tor review this interesting case report. In my opinion the subject is very interesting and report is useful for the diagnosis and management of such rare clinical event. 

 In my opinion an important point to be discussed is the possibility to preserve the placenta for 24-48 hours after each birth, to perform histological examination even in the case in which the birth was physiological but particular symptoms appeared in the hours immediately after birth (also in this case, after an in-depth medical history, the bleeding was already abundant after birth. This procedure could led to a better and faster diagnosis of this as well as other even rarer placental pathologies [ref 1] . A few more histological tests would improve the outcomes in terms of survival and fertility sparing of the patients.

Are enough details provided of any physical examination and diagnostic tests, treatment given and outcomes?

Yes

Is the case presented with sufficient detail to be useful for other practitioners?

Yes

Is sufficient discussion included of the importance of the findings and their relevance to future understanding of disease processes, diagnosis or treatment?

No

Is the background of the case’s history and progression described in sufficient detail?

Yes

Reviewer Expertise:

Expert in Placental Pathology

I confirm that I have read this submission and believe that I have an appropriate level of expertise to confirm that it is of an acceptable scientific standard, however I have significant reservations, as outlined above.

## References

[1] : Maternal and fetal outcomes of intraplacental choriocarcinoma complicated by fetomaternal hemorrhage: a systematic review. *J Matern Fetal Neonatal Med* .2023;36(2) : 10.1080/14767058.2023.2285238 2285238 10.1080/14767058.2023.2285238 38010764

# Reviewer report: Reviewer response for version 1

**DOI:** 10.5256/f1000research.78892.r159682

**Published:** 2023-01-19

**Authors:** Maria-Gabriela Aniţei

**Affiliations:** 1 University of Medicine and Pharmacy, Iasi, Romania

I was pleased to review the article **Case Report: Placental site trophoblastic tumor revealed by a clinical pelvic abscess.**

 The methodology used by the authors is appropriate for the purpose of the study and conclusions are narrowly linked to available evidence. The title expresses clearly the content of the manuscript and highlights the importance of the study. As the authors note in **Strengths and limitations**, this case report is interesting since it is the first reporting of PSTT simulating a pelvic abscess. There was rapid evolution of tumor development, only 2 months after natural birth, apparently without a risk factor from her medical history. There are interesting images intraoperative and microscopy

 The article respects the presentation algorithm that is required for a case report type article, but there is a number of medical details are missing, such as no detailed investigations (CT-scan, for example) to exclude metastases, both at the time of diagnosis and follow-up.

 In the same direction, to improve the methodology of this study, the introduction section must be improved. It is too short, and I suggest that some information regarding diagnosis and prognosis of PSTT should be included. Likewise, Immunohistochemistry data and the FIGO score are missing from the anatomopathological presentation, this information would increase the value of the article.

 Likewise, to be more convincing, the authors could image the patient at a distance through an CT-scan exam to confirm, through this high-performance investigation, the absence of local or distant disease. These tests would help to exclude metastases, both at the time of diagnosis and follow-up, especially as the anatomical-pathological examination shows a vascular invasion.

 The conclusion of the article could be strengthened. **Although PSTTs are extremely rare worldwide, the diagnosis should be considered during the post-partum and postabortion periods if there is persistent metrorrhagia** - this means that, in front of a patient with metrorrhagia and febrile syndrome that appeared early after delivery, this differential diagnosis (PSTT) must also be taken into account.

 Other points:

 1. It doesn't seem representative to me that **pathology **should be a Keywords

 2.  immunohistochemistry data and the FIGO score are missing from the anatomopathological presentation

 3. Usually, 'PSTTs are benign tumors' – I am not agree with this affirmation

 4. In figure 3- the microscopy details must be specified - is it an image of HE? , how many times is it amplified ( which objective was used?) . As the area of interest is identified in image D, I recommend that the same thing be done for the other images (Figure 3- A, B, C, E)

Are enough details provided of any physical examination and diagnostic tests, treatment given and outcomes?

Partly

Is the case presented with sufficient detail to be useful for other practitioners?

Partly

Is sufficient discussion included of the importance of the findings and their relevance to future understanding of disease processes, diagnosis or treatment?

Yes

Is the background of the case’s history and progression described in sufficient detail?

Partly

Reviewer Expertise:

oncological gynecology

I confirm that I have read this submission and believe that I have an appropriate level of expertise to state that I do not consider it to be of an acceptable scientific standard, for reasons outlined above.

# Reviewer report: Reviewer response for version 1

**DOI:** 10.5256/f1000research.78892.r125758

**Published:** 2022-03-07

**Authors:** Antonio Braga

**Affiliations:** 1 Rio de Janeiro Trophoblastic Disease Center, Antonio Pedro University Hospital, Maternity School of Rio de Janeiro Federal University, Fluminense Federal University, Rio de Janeiro, Brazil

Dear Authors and Editors,

*I would like to thank you for the opportunity to review the paper entitled: “Case Report: Placental site trophoblastic tumor revealed by a clinical pelvic abscess”, submitted to the F1000. Similarly, I would like to congratulate the authors for their interest in the topic.*

 In this case report, the authors presented a description of a PSTT presenting as abdominal abscess, reviewing the literature of this subject.

 I would like to highlight some aspects that deserve attention.

1. The authors must describe in the case report the screening for metastatic disease, to justify the decision for TAH.
2. PSTT is always cancer, not a benign tumor as described.
3. Nowadays we have, besides FIGO anatomical staging system as prognostic factor for PSTT surviving, the interval of last pregnancy and the initiating of treatment of 48 months. ^1^
4. In the section 'Strengths and Limitations' there is a PTTT that should be corrected.

Are enough details provided of any physical examination and diagnostic tests, treatment given and outcomes?

Partly

Is the case presented with sufficient detail to be useful for other practitioners?

Yes

Is sufficient discussion included of the importance of the findings and their relevance to future understanding of disease processes, diagnosis or treatment?

Yes

Is the background of the case’s history and progression described in sufficient detail?

Partly

Reviewer Expertise:

Gestational trophoblastic disease

I confirm that I have read this submission and believe that I have an appropriate level of expertise to confirm that it is of an acceptable scientific standard, however I have significant reservations, as outlined above.

## References

[1] : Intensified therapies improve survival and identification of novel prognostic factors for placental-site and epithelioid trophoblastic tumours. *Br J Cancer* .120(6) : 10.1038/s41416-019-0402-0 587-594 10.1038/s41416-019-0402-0 30792530 PMC6461960

# Response

**Published:** 2023-10-20

**Authors:** chaouki mbarki

**Affiliations:** Ben arous hospital, Faculty of medecine of Tunis university Tunis EL MANAR, Tunisia

Dear reviewer,

 Thank you for taking the time to review our article and for your valuable feedback. I will address your comments as follows:

 Regarding your first comment, the patient had undergone a total abdominal hysterectomy after a thoracoabdominopelvic CT scan revealed no metastatic lesions.

 We have also addressed all the other issues as per your request, which you can verify in the revised version.

 We would greatly appreciate it if you could approve this paper now that we have made the necessary amendments.

 Thank you for your assistance.
